# Scenario-based assessment of emergency management of urban infectious disease outbreaks

**DOI:** 10.3389/fpubh.2024.1368154

**Published:** 2024-04-24

**Authors:** Pengwei Yuan, Huifang Liu, Xiaoqing Dong

**Affiliations:** ^1^Business School, University of Jinan, Jinan, Shandong, China; ^2^College of Management and Economics, Tianjin University, Tianjin, China

**Keywords:** CIA-ISM, scenario approach, COVID-19, Wuhan, pestilence

## Abstract

Infectious diseases pose a severe threat to human health and are accompanied by significant economic losses. Studies of urban outbreaks of infectious diseases are diverse. However, previous studies have neglected the identification of critical events and the evaluation of scenario-based modeling of urban infectious disease outbreak emergency management mechanisms. In this paper, we aim to conduct an empirical analysis and scenario extrapolation using a questionnaire survey of 18 experts, based on the CIA-ISM method and scenario theory, to identify the key factors influencing urban infectious disease outbreaks. Subsequently, we evaluate the effectiveness of urban infectious disease outbreak emergency management mechanisms. Finally, we compare and verify the actual situation of COVID-19 in China, drawing the following conclusions and recommendations. (1) The scenario-based urban infectious disease emergency management model can effectively replicate the development of urban infectious diseases. (2) The establishment of an emergency command center and the isolation and observation of individuals exposed to infectious diseases are crucial factors in the emergency management of urban outbreaks of infectious disease.

## 1 Introduction

Among public health emergencies, infectious diseases have seriously endangered human health due to their suddenness, contagiousness, epidemic nature, and unpredictability, becoming a significant public health and social problem threatening people’s health and safety. Moreover, they can lead to a range of economic and social issues. Novel infectious diseases may result in severe human casualties, substantial economic losses, and other catastrophic consequences (1). For instance, COVID-19 is wreaking havoc globally. According to the latest statistics from Hopkins University, as of May 24, 2022, Beijing time, there were 523,086,544 cumulative confirmed cases of COVID-19 and 449,678,693 cumulative deaths worldwide. A report by the Asian Development Bank suggests that the global economic damage caused by COVID-19 amounts to 5.5–8.7% of global GDP in 2020 and 3.6–6.3% in 2021. The COVID-19 Updated Assessment of Potential Economic Impacts report indicates that global employment may decline by 158 million to 242 million jobs in 2021. Global labor income may decrease from $1.2 trillion to $1.8 trillion. Additionally, uncertainty surrounding novel viral infections and treatment raises public anxiety and psychological burden, leading to mass panic (2). Misinformation and unverified information regarding COVID-19 spread rapidly on social media and traditional media (3). These social issues also pose significant challenges to national and city emergency management (4).

SRAS, H_1_N_1_, and Ebola outbreaks have provided valuable lessons in managing infectious diseases. Lin et al. summarized emergency management procedures in radiology departments during the SARS outbreak (5). Fraser and Donnelly assessed the severity of H_1_N_1_ and emphasized the need for effective health measures to combat infectious diseases (6). Brooks et al. emphasized the importance of establishing a functional incident management system (IMS) during the Ebola virus disease outbreak in West Africa (7). Chavez, Long, et al. underscored the crucial role of physicians in controlling emerging infectious diseases in cities (8). In the early stages of a new infectious disease outbreak, it is essential to follow available guidelines and strictly adhere to infection control principles (9). Zhang and Zhou encourage maintaining social distance and implementing measures based on population characteristics after a new infectious disease outbreak (10). Glass and Glass proposed tailored social distancing measures for young people and children in infectious disease contexts (11). In the event of a shortage of public health facilities and medical resources, hospitals must allocate resources equitably based on the best health outcomes, guided by local government or professional bodies (12). Societal-level measures like social isolation, blockades, border closures, and human tracking can help control COVID-19 transmission (13–15). Scenarios are dynamic model forms of events that enable changing outcomes by controlling event likelihood, providing decision-makers with tools to synthesize trends and events into manageable options. They add value to traditional planning techniques (16). Previous studies overlooked the interaction between emergency management elements in reducing urban infectious disease impacts, neglecting scenario-based assessments of urban infectious disease outbreaks, despite their applicability to novel infectious diseases.

This paper aims to address two main questions: What are the relationships between key events in urban outbreak emergency management of infectious diseases, and how do these interactions impact the effectiveness of urban infectious disease emergency management across different scenarios? Additionally, how can practical and effective suggestions be provided for emergency management decision-makers? To tackle these issues, key events in urban infectious disease outbreaks were identified. The paper employs the CIA, ISM, and Delphi methods to establish a scenario-based assessment model for urban infectious disease emergency management. Subsequently, interactions were analyzed, and urban emergency management was simulated under various scenarios to assess the impact of emerging infectious diseases on economic loss, human casualties, disease control, and public trust. Finally, an evaluation of Wuhan, China’s emergency management response to COVID-19, an urban emerging infectious disease, was conducted. The contributions of this paper compared to existing studies include: (1) Extraction of key events for emergency management of emerging urban infectious diseases and clarification of interrelationships among actual events related to urban infectious diseases. (2) Proposal of an integrated model for scenario analysis by combining the CIA, ISM, and Delphi methods. (3) The model’s ability to evaluate the effectiveness of urban infectious disease emergency management and provide proven recommendations for decision-makers through scenario analysis and extrapolation of urban infectious disease development.

## 2 Literature review

Research on emergency management of infectious diseases has primarily focused on preparedness, transmission modeling, economic loss assessment, and the societal impact of outbreaks. Brouqui et al. developed an infectious disease control framework for managing outbreaks (17). Wong et al. examined emergency preparedness for SARS and avian influenza in Hong Kong and proposed new public health management measures (18). The CDC has implemented measures to address EBOV spread, including monitoring travelers from affected areas and assessing hospital capacities (19). Effective collaboration among medical personnel and sufficient medical resources are crucial in emergency management. Therefore, it is essential to provide a safe working environment, adequate food, rest, and psychological support for healthcare workers globally (20). Lam et al. analyzed the challenges of guideline implementation and emergency management in acute care settings (21). Huang et al. outlined emergency management and infection control strategies in the radiology department, resulting in zero COVID-19 infections among staff (22).

In developing infectious disease transmission models, Kucharski et al. used SARS-CoV-2 transmission data from Wuhan in 2019 to show that regional closures reduce transmission, but delayed closures may lead to widespread outbreaks (23). Wu and Leung utilized exported case data to model outbreaks in major Chinese cities, indicating inevitable global outbreaks in urban centers (24). Razavi-Shearer et al. estimated regional HBV prevalence using a dynamic transmission model in the context of childhood vaccination (25). Chang et al. simulated SARS-CoV-2 spread in 10 United States cities, highlighting mobility’s role in increased infection rates and economic losses (26). Musa et al. modeled COVID-19 transmission and mortality, suggesting that a combination of social distancing and vaccination could reduce mortality in South America (27).

COVID-19 poses significant threats to economic development, social systems, and human life, instigating concerns about potential economic crises or recessions. Kim and Loayza argue that, compared to high-income countries, the benefit governments in low-income nations gain from not intervening in epidemic prevention measures is marginal (28). Maria and Zaid Alsafi provide an overview of COVID-19’s impact on various aspects of the global economy (1, 29).

The array of social crises resulting from infectious diseases should not be underestimated, with panic being a prevalent psychological response to new outbreaks. How governments assess and address public panic can significantly influence the effectiveness of infectious disease emergency management mechanisms (30). A panic-induced rush for supplies is highly probable, underscoring the importance of maintaining adequate supplies during significant public health events (31, 32). Wang et al. utilized the DASS-21 model to evaluate the psychological status of 1738 individuals in China, emphasizing the need to focus on case escalation, promote proper coping mechanisms, and enhance personal protection in response to COVID-19 (33). Rumors often stem from panic, hindering emergency management efforts by spreading misinformation (34). Hui et al. developed a rumor propagation model based on infectious disease models, suggesting effective strategies to counteract the spread of COVID-19 rumors (35). Ning et al. underscore the importance of authoritative announcements in dispelling rumors and mitigating their impact during the early months of the COVID-19 outbreak in China (36). Individuals seek to minimize losses and avert catastrophic consequences through pivotal decisions (37).

While most studies have examined the impact of individual events or factors, emergency management responses to a single factor may prove less effective when multiple events or factors interact. To ensure reliable and effective emergency management of urban COVID-19 outbreaks, management strategies must consider the cross-influences among various factors. The scenario-based approach assesses interactions between critical events within a specified timeframe (38), enhancing managers’ understanding of the factors involved in developing COVID-19 contingency plans and the response measures required. When integrated with other predictive models, scenario models offer flexible approaches to address uncertainty. This paper examines potential future trends in urban infectious disease outbreaks, identifies key factors, and proposes optimal decisions.

## 3 Research methodology

### 3.1 Basis of the scenario approach

The scenario-based emergency management model for urban outbreaks of infectious diseases combines the Delphi method, the cross-influence approach (CIA), and the Interpretative Structural Model (ISM). Its purpose is to analyze scenarios and interpret the progression of urban infectious disease outbreaks. The Delphi method and CIA enable the analysis of factors influencing urban outbreaks. CIA identifies relationships between events affecting outcomes, enhancing the stability of identified events. However, using CIA requires presenting a series of interrelated future events. A team of experts conducts a Delphi process to predict event occurrence based on inputs, jointly assessing cross-impacts.

### 3.2 Creation of event set

Creating the COVID-19 event set is based on observing and studying historical infectious disease cases. Addressing significant health events necessitates considering technical and social factors and obtaining input from experts with diverse experiences and perspectives. This approach aims to identify critical factors and relationships, ensuring decision-making rationality, internal consistency, and usefulness. A comprehensive set of 32 relevant events was selected and categorized into three types. Initial events (IC_i_): These events occur or not before infectious disease outbreaks. They may reflect emergency management of urban outbreaks, significantly impacting disease development. Experts subjectively estimate initial event probabilities at 0.5. If an event with a probability below 0.5 is anticipated, experts reassess other events’ probabilities.

IC_1_: Infectious disease isolation and treatment capacity: The city can isolate and treat patients with infectious diseases.

IC_2_: Infectious disease source detection capability: The city can quickly conduct rapid detection of the cause of the disease.

IC_3_: Infectious disease infectivity assessment: The city can quickly assess the infectious characteristics or transmission routes of infectious diseases.

IC_4_: Medical treatment.

IC_5_: Government emergency response plan: The city has an excellent emergency response plan for infectious diseases.

IC_6_: Government emergency response capability: The city has an infectious disease control agency, conducts frequent emergency drills, and has good response capability.

IC_7_: Public self-protection ability: The public knows the general knowledge of infectious diseases and has good self-protection ability.

IC_8_: Government communication capability: Have internal communication procedures and public communication plans and channels.

IC_9_: Public trust: The public trusts the government and complies with government emergency instructions.

IC_10_: Vaccine: There is no vaccine for infectious disease.

Dynamic events (DE_i_) encompass post-epidemic occurrences, primarily consisting of government emergency management measures and events triggering secondary disasters. These events are assigned a probability of occurrence of 0.5.

DE_1_: Infectious disease patient treatment: Infectious disease patients are isolated and treated.

DE_2_: Causal cause identification: The infectious disease causative agent (bacteria, virus) is rapidly identified.

DE_3_: Infectious disease transmissibility assessment: The transmission route and transmissibility of infectious diseases are rapidly assessed.

DE_4_: Decontamination action: Disinfect the patient’s activity area after infection.

DE_5_: Isolate the contacts: Isolate and observe people who are exposed to infectious diseases.

DE_6_: Leaders do not agree to release information: Local heads do not agree to inform the public about the status of the infectious disease.

DE_7_: Information leak: Social media leaks information about the infectious disease to the public.

DE_8_: Emergency command center established: The government establishes an emergency command center and declares a state of emergency.

DE_9_: Public panic: The public panics and either rushes to buy supplies or leaves the city by transportation if possible.

DE_10_: Rumors spread: Various rumors appear on the Internet and social software.

DE_11_: Spread of infectious diseases: Infectious diseases spread among the population.

DE_12_: Restriction of movement of people: The government restricts the movement and gathering of people.

DE_13_: Communicating infectious diseases: The government communicates about infectious diseases through the media and widely publicizes the hazards and protection. The government calls on and organizes businesses, non-profit organizations, and individuals for infectious disease prevention and control.

DE_14_: Emergency medical supplies are distributed to organizations and people in need promptly.

DE_15_: Some healthcare workers do not cooperate: Some healthcare workers or emergency managers refuse to work on infectious diseases because of a lack of protective materials or fear of being infected.

DE_16_: Some members of the public do not cooperate: Some people do not comply with government measures for epidemic prevention.

DE_17_: Area closure: Other cities restrict the entry of people from that city, and people from that city are not allowed to leave the city.

DE_18_: Development of effective drugs/vaccines: Effective types of existing drugs are proposed, vaccines are developed, and mass production is carried out.

Outcome event (OE_i_) encompasses various consequences that may arise from an infectious disease event, such as casualties and property damage. The probability of an outcome event is determined accurately at the end of the period, with an initial probability also set to 0.5.

OE_1_: Economic loss: GDP loss caused by infectious diseases.

OE_2_: Personnel Casualties: a certain number of infectious disease patients die.

OE_3_: Infectious disease is controlled: The city’s Infectious disease is controlled in a phased manner, and the number of sick people gradually decreases without spreading to other cities or regions.

OE_4_: Public trust: The public has a high level of trust in the local government after the epidemic and continues cooperating with the government in its actions.

Expert estimates of the relationship between the three-event sets (initial conditions, dynamic events, and outcome events) were sought.

### 3.3 Scenario analysis

**Figure 1:**
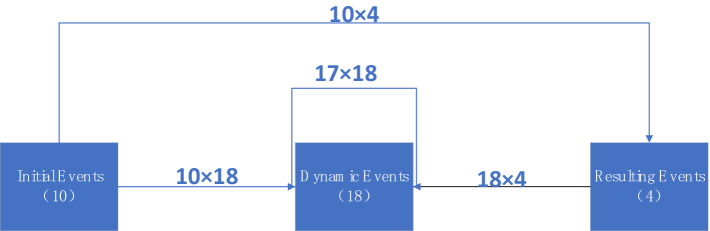
Influence diagram of the number of three event sets and the number of estimates required.

Eighteen experts in emergency management and frontline rescue were invited to participate in the expert panel. They assessed whether each of the three event sets occurred and estimated the probability of other events happening. Due to the diverse nature of each event, the panel had to make 478 causality estimates, as illustrated in Figure 1.

Events within a set are interconnected. However, in the Delphi method, the occurrence or non-occurrence of one event does not impact others in the set. Therefore, we integrated the CIA into the ISM and used it to obtain the three event sets of COVID-19 as input for the ISM. The most critical aspect of CIA-ISM is “structural modeling.” The ISM approach provides a solid mathematical foundation for establishing linear relationships between critical events and their influencing factors. Initially, experts estimate the relationships and probabilities between events. Then, a model is constructed to reflect the complex relationships over time, with the flexibility to receive feedback at different stages, enabling experts to adjust inputs as needed.

The occurrence probability of event i, influenced by event j, follows the rule outlined in Table 1, known as the scoring table. A valid factor metric between i and j is established when at least two-thirds of the individual interaction estimates fall within an interval in the scoring table. The resulting adjacency matrix serves as input for the CIA, where cells represent the linear influence factor of event i on j, denoted as R_ij_. Diagonal cells indicate the total probability. Using this binary matrix, we can forecast scenarios by assuming event i’s occurrence (or non-occurrence).

**Table 1:** Rating scale.

Number	Explanation
0.99	Significant positive impact.
0.9	Apparent positive impact.
0.8	Great positive impact.
0.7	Modest positive impact.
0.6	Slight positive impact.
0.5	No impact.
0.4	Slight negative impact.
0.3	Modest negative impact.
0.2	Great negative impact.
0.1	Apparent negative impact.
0.01	Significant negative impact.

### 3.4 Cross-impact analysis

In Table 1, a mathematical “+” denotes event facilitation, while a mathematical “-” signifies suppression. These symbols only show the impact’s direction and do not denote magnitude. Equations 1, 2 represents the cross-influence factor and event occurrence probability.


(1) $$C_{ij}=\frac{1}{1-P_{j}}\left\{\left[\ln\frac{R_{ij}}{\left(1-R_{ij}\right)}\right]-\left[\ln\frac{P_{i}}{\left(1-P_{i}\right)}\right]\right\}$$

(2) $$P_{i}=\frac{1}{1+\exp\left(-G_{i}-\sum_{k\neq i}^{N}C_{ik}P_{k}\right)}$$

where G_i_ is the sum of the effects of all possible external events on the event i that are not explicitly represented in the n events included in the model. To obtain a numerical estimate of the total variability in the matrix, we examined the following linear sum of the cross-impact factorΣ|C_ij_|.

|internal event impact| = Σ|C_ij_| = 683.8245761.

|initial event impact| = Σ|C_ij_| = 293.4260838.

|Dynamic event impact| = |internal event impact|-|initial event impact| = Σ|C_ij_|-Σ|C_ij_| = 535.9788616.

|External unspecified event impact| = Σ|C_ij_| = 186.593908.

|Total impact| = |internal event impact| + |external unspecified event impact| = Σ|C_ij_| + Σ|C_ij_| = 870.4184841.

Calculate the relative fraction or percentage of impact due to each type of event.

|Dynamic event impact|/|Total impact| = 0.615771461.

|initial event impact|/|total impact| = 0.337109206.

|External unspecified event impact|/|Total impact| = 0.21437264.

Thus, unspecified events (external event impact) contribute to 21.44% of the overall impact, while dynamic events and initial conditions contribute 61.58 and 33.71%, respectively. In total, 78.56% of the impact in the model is attributed to explicit events. This suggests that the event set is relatively comprehensive, rendering the model feasible.

After deriving the cross-impact matrix, we delineate the strong relationships within the directed graph model by partitioning and extracting the matrix model. By employing the CIA-ISM method, we can illustrate the anticipated scenario using a directed graph. Table 2 outlines the direct and indirect effects of each event on the outcome events (cascade effects). The model suggests that event DE_1_ may either positively impact favorable outcomes or negatively influence adverse outcomes. IC_6_, IC_8_, and IC_9_ directly negatively impact DE_15_, indicating that government emergency response capacity, establishment of emergency command centers, and public trust can significantly decrease the likelihood of partial health care worker non-cooperation. Similarly, IC_2_, IC_7_, and DE_2_ directly negatively influence DE_16_, signifying that enhancing infectious disease source detection capabilities, information disclosure, and causation identification would markedly diminish the probability of partial public non-cooperation.

**Table 2:** Outcome events analysis.

Events	-OE_1_,-OE_2_ +OE_3_,+OE_4_	+OE_1_,+OE_2_ −OE_3_,−OE_4_
IC_1_:Infectious disease isolation and treatment capacity. IC_2_:Infectious disease source detection cap-ability. IC_3_:Infectious disease infectivity assessment. IC_4_:Medical treatment. IC_5_:Government emergency response plan. IC_6_:Government emergency response capability. IC_7_:Public self-protection ability. IC_8_:Government communication capability. IC_9_:Public trust. IC_10_:Vaccine. DE_1_:Infectious disease patient treatment. DE_2_:Causal cause identification. DE_3_:Infectious disease transmissibility assessment. DE_4_:Decontamination action DE_5_:Isolate the contacts DE_6_:Leaders do not agree to release information DE_7_:Information leak DE_8_:Emergency command center established DE_9_:Public panic DE_10_:Rumors spread DE_11_:Spread of infectious diseases DE_12_:Restriction of movement of people DE_13_:Communicating infectious diseases DE_14_:Emergency medical supplies are distributed to organizations and people in need promptly. DE_15_:Some healthcare workers do not cooperate DE_16_:Some members of the public do not cooperate DE_17_:Area closure DE_18_:Development of effective drugs/vaccines	+ + + + + + + + + + + + + + – – + – – – + + + – – + + +	– – – – – – – – – – – – – – + + – + + + – – – + + – – –

Among all emergency response efforts, the establishment of an emergency command center (DE_8_) has a direct and significant impact on reducing economic losses (OE_1_), controlling infectious diseases (OE_3_), and increasing public trust (OE_4_). Limiting the movement of people (DE_12_) made a considerable contribution to increasing public trust (OE_4_). Among all secondary-derived disasters, the spread of infectious diseases (DE_11_) directly and significantly impacts human casualties (OE_2_).

Analyzing outcome events can be enhanced by considering the direct impact of initial conditions and dynamic events on these specific outcomes, as shown in Tables 3–6. For instance, Table 3 demonstrates that the model anticipates leadership disagreement in releasing information, while partial public non-cooperation emerges as the primary precursor to potential economic loss. Conversely, establishing an emergency command center, evaluating infectious disease transmission capacity, and implementing a government contingency plan markedly diminish the probability of such an outcome.

**Table 3:** OE_1_ ordered influences table.

	Events	OE_1_
DE_8_	Emergency command center established.	3.4692
IC_5_	Government emergency response plan.	2.9
IC_3_	Infectious disease infectivity assessment.	2.7726
DE_3_	Infectious disease infectivity assessment.	2.3054
IC_8_	Government communication capability.	2.1972
DE_12_	Restriction of movement of people.	1.8889
DE_17_	Area closure.	1.8889
DE_4_	Decontamination action.	1.6002
DE_5_	Isolate the contacts.	1.3266
IC_4_	Medical treatment.	0.8946
DE_2_	Causal cause identification.	0.8946
IC_1_	Infectious disease isolation and treatment capacity.	0.8109
DE_13_	Communicating infectious diseases.	0.7279
IC_6_	Government emergency response capability.	0.5637
IC_2_	Infectious disease source detection capability.	0.4013
DE_1_	Infectious disease patient treatment.	0.3207
IC_7_	Public self-protection ability.	0.2403
DE_14_	Emergency medical supplies are distributed to organizations and people in need promptly.	0.2403
DE_15_	Some healthcare workers do not cooperate.	−0.16
DE_11_	Spread of infectious diseases.	−0.401
DE_18_	Development of effective drugs/vaccines.	−1.064
IC_10_	Vaccine.	−1.889
DE_6_	Leaders do not agree to release information.	−2.417
DE_16_	Some members of the public do not cooperate.	−2.9

**Table 4:** OE_2_ ordered influences table.

	Events	OE_2_
DE_8_	An emergency command center was established.	2.4166
DE_5_	Isolate the contacts.	2.0919
IC_8_	Government communication capability.	1.7908
DE_4_	Decontamination action.	1.3266
IC_3_	Infectious disease infectivity assessment.	1.2381
IC_4_	Medical treatment.	1.2381
DE_12_	Restriction of movement of people.	1.2381
DE_3_	Infectious disease infectivity assessment.	1.1507
IC_6_	Government emergency response capability.	1.0644
IC_2_	Infectious disease source detection capability.	0.8109
DE_14_	Emergency medical supplies are distributed to organizations and people in need promptly.	0.5637
IC_5_	Government emergency response plan.	0.4013
DE_13_	Communicating infectious diseases.	0.4013
DE_1_	Infectious disease patient treatment.	0.3207
DE_2_	Causal cause identification.	0.2403
IC_7_	Public self-protection ability.	0.2403
IC_1_	Infectious disease isolation and treatment capacity.	0.1601
DE_15_	Some healthcare workers do not cooperate.	−0.16
DE_11_	Spread of infectious diseases.	−0.401
DE_18_	Development of effective drugs/vaccines.	−0.646
IC_10_	Vaccine.	−1.889
DE_6_	Leaders do not agree to release information.	−2.417
DE_16_	Some members of the public do not cooperate.	−2.9

**Table 5:** OE_3_ ordered influences table.

	Events	OE_3_
DE_11_	Spread of infectious diseases.	3.1713
IC_10_	Vaccine.	2.0919
DE_16_	Some members of the public do not cooperate.	1.5075
DE_18_	Development of effective drugs/vaccines.	1.3266
DE_15_	Some healthcare workers do not cooperate.	1.1507
DE_10_	Rumors spread.	1.0644
DE_6_	Leaders do not agree to release information.	0.8109
DE_7_	Information leak.	0.8109
IC_1_	Infectious disease isolation and treatment capacity.	−0.16
IC_2_	Infectious disease source detection capability.	−0.321
IC_4_	Medical treatment.	−0.482
DE_3_	Infectious disease infectivity assessment.	−0.482
IC_6_	Government emergency response capability.	−0.564
DE_17_	Area closure.	−0.564
IC_5_	Government emergency response plan.	−1.064
IC_8_	Government communication capability.	−1.889
DE_14_	Emergency medical supplies are distributed to organizations and people in need promptly.	−1.989
IC_9_	Public trust.	−2.092
DE_13_	Communicating infectious diseases.	−2.305
IC_3_	Infectious disease infectivity assessment.	−2.417
DE_2_	Causal cause identification.	−2.417
DE_1_	Infectious disease patient treatment.	−2.65
DE_5_	Isolate the contacts.	−2.65
IC_7_	Public self-protection ability.	−2.773
DE_4_	Decontamination action.	−2.773
DE_12_	Restriction of movement of people.	−2.773
DE_8_	An emergency command center was established.	−3.631

**Table 6:** OE_4_ ordered influences table.

	Events	OE_4_
DE_9_	Public panic.	3.0327
DE_11_	Spread of infectious diseases.	2.7726
DE_18_	Development of effective drugs/vaccines.	2.7726
DE_10_	Rumors spread.	1.4164
DE_6_	Leaders do not agree to release information.	0.2403
IC_8_	Government communication capability.	−0.16
IC_6_	Government emergency response capability.	−0.24
IC_4_	Medical treatment.	−0.321
DE_14_	Emergency medical supplies are distributed to organizations and people in need promptly.	−0.728
IC_9_	Public trust.	−1.508
DE_12_	Restriction of movement of people.	−1.6
IC_5_	Government emergency response plan.	−1.889
DE_13_	Communicating infectious diseases.	−1.989
IC_3_	Infectious disease infectivity assessment.	−2.092
IC_1_	Infectious disease isolation and treatment capacity.	−2.305
DE_7_	Information leak.	−2.417
DE_3_	Infectious disease infectivity assessment.	−2.531
IC_2_	Infectious disease source detection capability.	−2.65
DE_2_	Causal cause identification.	−3.171
DE_8_	An emergency command center was established.	−4.181

We enumerate the events that directly influence the outcome event, whether positively or negatively. Conversely, critical factors influencing the containment of infectious diseases and fostering public trust included establishing an emergency command center, identifying the causative agent, and limiting people’s movement (see Tables 5, 6). Conversely, factors such as infection spread, public panic, and vaccine unavailability hindered these desired outcomes.

Tables 3–6 present the key events influencing the occurrence or absence of each of the four outcomes. The values in Table 7 are calculated by summing the absolute positive impacts of C_ij_ on the events. Table 8 displays the most significant events contributing to adverse outcomes. The weights presented indicate the cumulative adverse impact, represented by the sum of the absolute negative impacts of C_ij_ on the events.

**Table 7:** Total impact on positive events.

	Events	OE_3_,OE_4_
DE_8_	An emergency command center was established.	7.812062127
DE_11_	Spread of infectious diseases.	5.94384325
DE_2_	Causal cause identification.	5.587876939
IC_3_	Infectious disease infectivity assessment.	4.508559522
DE_12_	Restriction of movement of people.	4.372827322
DE_13_	Communicating infectious diseases.	4.29460417
DE_18_	Development of effective drugs/vaccines.	4.099177157
IC_9_	Public trust.	3.599480715
DE_7_	Information leak.	3.227552628
DE_9_	Public panic.	3.032694979
DE_3_	Infectious disease infectivity assessment.	3.01365686
IC_2_	Infectious disease source detection capability.	2.97053613
IC_5_	Government emergency response plan.	2.953356845
IC_7_	Public self-protection ability.	2.772588722
DE_4_	Decontamination action.	2.772588722
DE_14_	Emergency medical supplies are distributed to organizations and people in need promptly.	2.717175905
DE_1_	Infectious disease patient treatment.	2.649850829
DE_5_	Isolate the contacts.	2.649850829
DE_10_	Rumors spread.	2.480803743
IC_1_	Infectious disease isolation and treatment capacity.	2.465444435
IC_10_	Vaccine.	2.09193711
IC_8_	Government communication capability.	2.049008633
DE_16_	Some members of the public do not cooperate.	1.507543605
DE_15_	Some healthcare workers do not cooperate.	1.15072829
DE_6_	Leaders do not agree to release information.	1.05121884
IC_6_	Government emergency response capability.	0.803990928
IC_4_	Medical treatment.	0.803009414
DE_17_	Area closure.	0.563702304

**Table 8:** Total impact on negative events.

	Events	OE_1_,OE_2_
DE_8_	An emergency command center was established.	5.885824523
DE_11_	Spread of infectious diseases.	4.795790546
IC_10_	Vaccine.	4.30554563
IC_3_	Infectious disease infectivity assessment.	4.010667139
IC_8_	Government communication capability.	3.987992671
DE_6_	Leaders do not agree to release information.	3.481056039
DE_3_	Infectious disease infectivity assessment.	3.45608731
DE_5_	Isolate the contacts.	3.418525545
IC_5_	Government emergency response plan.	3.301361742
DE_12_	Restriction of movement of people.	3.127001634
DE_4_	Decontamination action.	2.926827035
DE_16_	Some members of the public do not cooperate.	2.900020351
IC_7_	Public self-protection ability.	2.545647644
IC_4_	Medical treatment.	2.132702853
DE_17_	Area closure.	1.888923218
DE_18_	Development of effective drugs/vaccines.	1.709980412
IC_6_	Government emergency response capability.	1.628135932
IC_2_	Infectious disease source detection capability.	1.212271607
DE_2_	Causal cause identification.	1.13491306
DE_13_	Communicating infectious diseases.	1.129272145
IC_1_	Infectious disease isolation and treatment capacity.	0.971015632
DE_14_	Emergency medical supplies are distributed to organizations and people in need promptly.	0.803990928
DE_1_	Infectious disease patient treatment.	0.480770715
DE_15_	Some healthcare workers do not cooperate.	0.400374039

### 3.5 Incremental analysis

**Figure 2:**
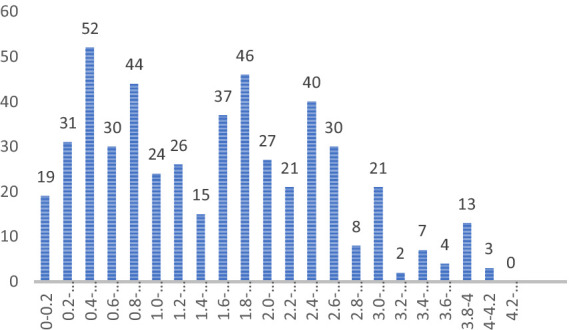
Histogram of |C_ij_|.

We conduct incremental analysis on the predicted scenarios to comprehend the relationships among different events. The primary approach involves analyzing the distribution of |C_ij_|. Non-zero |C_ij_| values are selected, and a histogram illustrating their frequency from zero to the maximum absolute value is plotted, as depicted in Figure 2.

**Figure 3:**
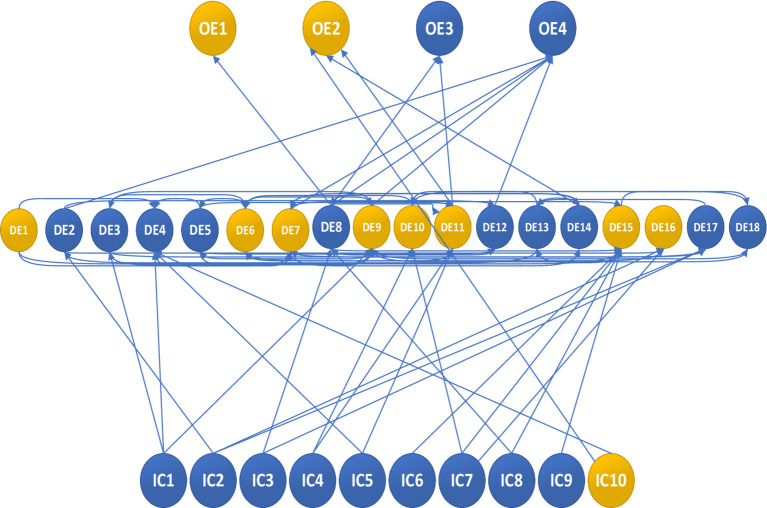
Digraph for the limit value |Cij| = 2.93 – with 15%.

**Figure 4:**
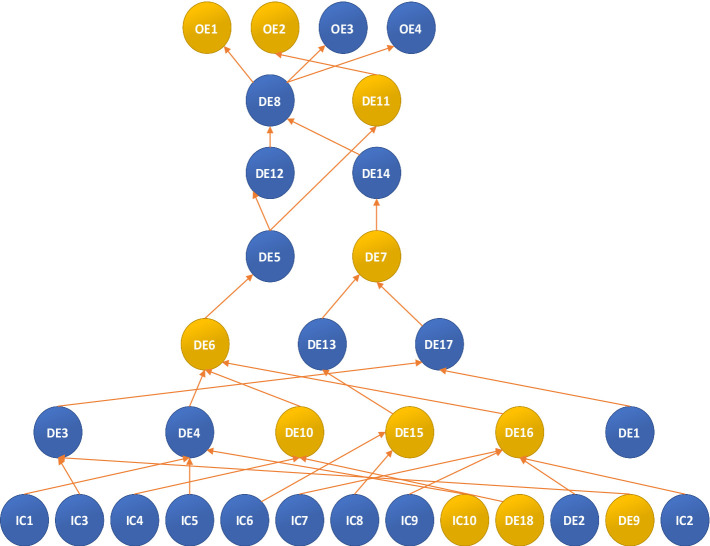
Digraph for the limit value |C_ij_| = 3.42 – with 5%.

Subsequently, we identify the value of |C_ij_| representing the highest k% of the distribution as the cut-off point for the directed graph. For instance, selecting 90 and 85% as the cut-off points results in the directed graph highlighting the top 10 and 15% impacts, as illustrated in Figures 3, 4. Lines connecting events of the same color denote positive impacts, while those connecting events of different colors signify negative impacts.

**Figure 5:**
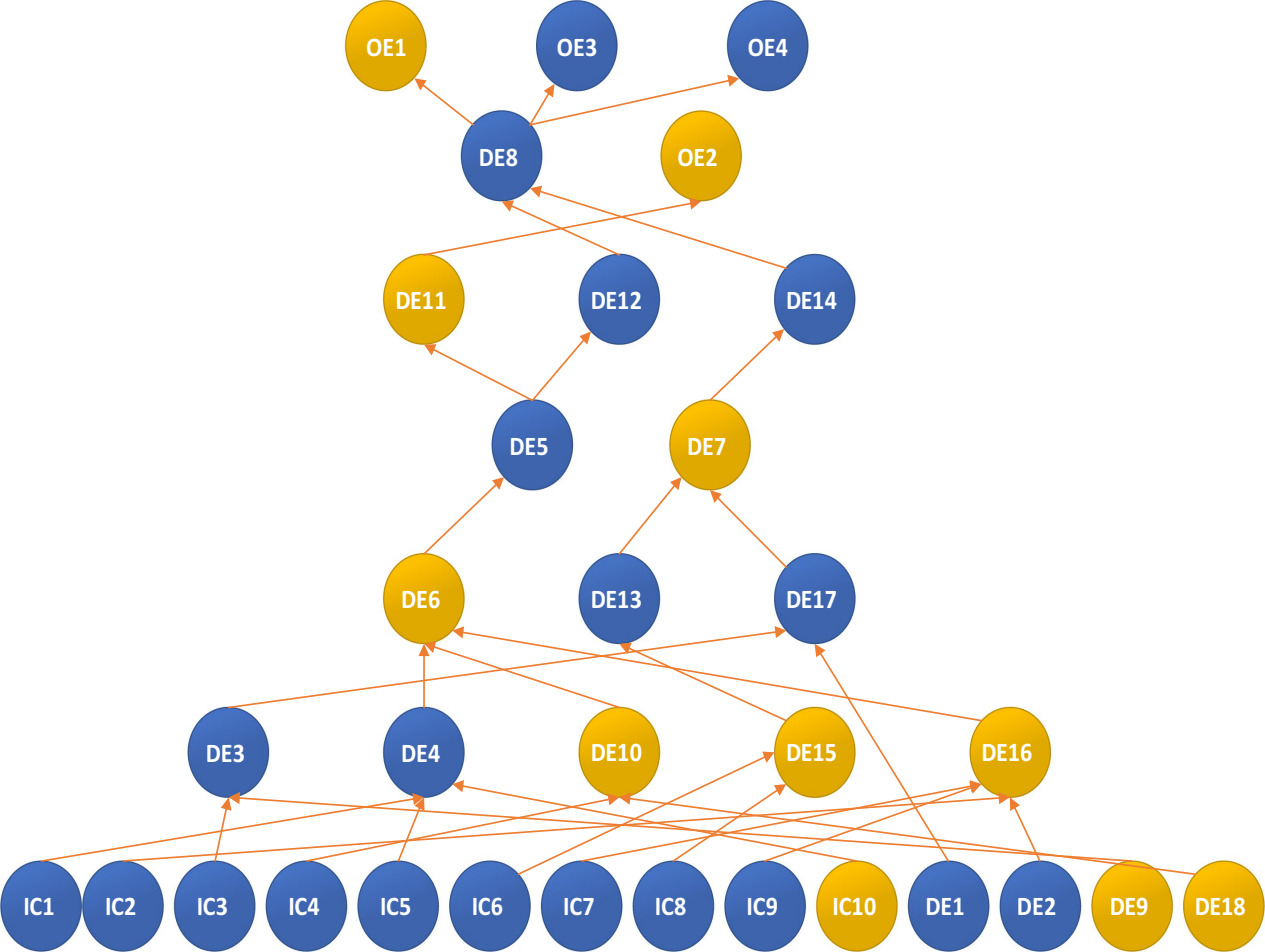
Digraph for the limit value |C_ij_| = 3.42 – with 5%.

The graph is directed, with the highest 85% of the distribution value determining its structure. At this analytical level, while all events are included, the logical sequence of events is not organized. To refine the model, additional |C_ij_| factors were integrated into the analysis, considering all |C_ij_| values greater than or equal to this threshold to establish the final model. At this stage, the |C_ij_| value of event i is utilized before or after the occurrence and non-occurrence of event j. The histogram of cross-influencing factors indicates that the limit value is |C_ij_| = 3.42 when extracting the most significant influence of the top 5%. Subsequently, the CIA-ISM output is displayed in Figure 4, with its adversarial plot depicted in Figure 5. If the value of |C_ij_| equals or surpasses the limit value, a direct connection from node j to node i is established. Identifying the most critical event in the event set through the limit value aids in comprehending the underlying logic of the particular influence path and scenario leading to that outcome, while also facilitating an understanding of the event sequence and its potential impact.

The figure illustrates that vaccines (IC_10_), capacity for infectious disease isolation and treatment (IC_1_), medical resource reserves (IC_4_), government contingency plans (IC_5_), public trust (IC_9_), government emergency response capacity (IC_6_), government communication capacity (IC_8_), public self-protection (IC_7_), infectious disease source detection (IC_2_), and assessment of infectious disease transmission capacity (IC_3_) trigger a series of dynamic events (DE_4_, DE_9_, DE_14_, DE_8_, DE_15_, DE_13_, DE_7_, DE_6_, DE_16_, DE_5_, DE_11_), which decrease the likelihood of death (OE_2_) and increase the chances of infectious disease control (OE_3_) and public trust (OE_4_). Prompt treatment of infected patients (DE_1_) directly limits movement restrictions (DE_17_), while vaccine shortages (IC_10_) negatively impact decontamination efforts (DE_4_), emphasizing the need for active vaccine development within decontamination measures (DE_4_). Enhancing the capacity for isolating and treating infectious diseases (IC_1_), developing government contingency plans (IC_5_), and creating effective vaccines against novel viruses (IC_10_) will greatly benefit decontamination operations (DE_4_). Insufficient medical resource reserves (IC_4_) and the absence of effective vaccines against novel viruses (IC_1_8) will lead to the spread of rumors (DE_1_0). Reduced public panic (DE_9_) and low infectious disease transmission capacity (IC_3_) would significantly improve the assessment of infectious disease transmission capacity (DE_3_). Strong public trust (IC_9_), high levels of infectious disease source detection (IC_2_), effective causative agent identification (DE_2_), and improved public self-protection (IC_7_) will notably decrease the likelihood of partial public non-cooperation (DE_16_). Effective government emergency response (IC_6_) and communication (IC_8_) will effectively mitigate non-cooperation among healthcare workers (DE_15_). Ineffective decontamination operations (DE_4_), partial public cooperation (DE_16_), and rumor dissemination (DE_10_) positively influence leaders’ consent to release information (DE_6_), suggesting that efficient decontamination operations (DE_4_), reduced rumor dissemination (DE_10_), and partial public non-cooperation (DE_16_) will likely prevent leaders from withholding information (DE_6_). Assessing infectious disease transmission capacity (DE_3_) and treating infectious disease patients (DE_1_) directly impact regional closure (DE_17_), whereas partial non-cooperation among healthcare workers (DE_15_) directly affects infectious disease communication (DE_13_). Disagreement among leaders regarding information release (DE_6_) directly hinders the implementation of contact isolation (DE_5_). Regional closure (DE_17_) and disease communication (DE_13_) directly decrease the likelihood of information leakage (DE_7_). Information leakage (DE_7_) significantly hampers the timely distribution of emergency medical supplies to those in need (DE_14_). Contact isolation (DE_5_) not only directly restricts people’s movement (DE_12_) but also acts as a deterrent to the spread of infectious diseases (DE_11_). Restricting people’s movement (DE_12_) and timely distribution of emergency medical supplies (DE_14_) facilitate the establishment of an emergency command center (DE_8_), which, in turn, increases the likelihood of disease control (OE_3_), public trust (OE_4_), and reduces economic losses (OE_1_). The diminished impact of contact isolation (DE_5_) on disease spread (DE_11_) directly decreases the likelihood of personnel casualties (OE_2_).

### 3.6 Sensitivity analysis

This paper aims to model the emergency management of urban infectious disease outbreaks, so the associated events are selected for sensitivity analysis. The impact of critical factors is tested by varying the initial probabilities.

#### 3.6.1 Initial conditions analysis

Based on the preceding analysis, it is evident that IC_4_, _5_, _6_, and _8_ are events pertinent to emergency management. Sensitivity analysis scrutinizes the outcomes of other events, particularly outcome events, by modifying the initial probabilities of these four events. We establish six scenarios to forecast occurrences where all four crucial events manifest, all fail to occur, and only one materializes. The initial probability of other events is set at 0.5. Utilizing formula (1, 2), we derive the probability of other events occurring across the six scenarios, as presented in Table 9. Economic loss (OE_1_) is influenced by the initial events in the descending order of IC_6_, IC_5_, IC_8_, and IC_4_. The government’s emergency response capability (IC_6_) plays a pivotal role in mitigating economic loss.

**Table 9:** Prediction probabilities of the other events in initial conditions analysis.

	S_0_	S_1_	S_2_	S_3_	S_4_	S_5_
IC_1_	0.5	0.5	0.5	0.5	0.5	0
IC_2_	0.5	0.5	0.5	0.5	0.5	0.5
IC_3_	0.5	0.5	0.5	0.5	0.5	0.5
IC_4_	0	1	0	0	0	1
IC_5_	0	0	1	0	0	1
IC_6_	0	0	0	1	0	1
IC_7_	0.5	0.5	0.5	0.5	0.5	0.5
IC_8_	0	0	0	0	1	1
IC_9_	0.5	0.5	0.5	0.5	0.5	0.5
IC_1_0	0.5	0.5	0.5	0.5	0.5	0.5
DE_1_	0.0012	0.2243	0.5124	0.7823	0.4124	0.9968
DE_2_	0.0015	0.3154	0.5239	0.7577	0.4239	0.9868
DE_3_	0.0017	0.2019	0.5287	0.8424	0.4287	0.9749
DE_4_	0.002	0.2534	0.5183	0.7676	0.4183	0.9821
DE_5_	0.0024	0.2314	0.5435	0.7733	0.4435	0.9677
DE_6_	0.8968	0.7754	0.4322	0.4134	0.6322	0.0012
DE_7_	0.8868	0.7655	0.4423	0.4234	0.6423	0.0015
DE_8_	0.0026	0.2412	0.5634	0.7324	0.3398	0.9135
DE_9_	0.9923	0.7123	0.4512	0.2243	0.6923	0.0012
DE_10_	0.8725	0.7612	0.4678	0.3154	0.6725	0.0015
DE_11_	0.9736	0.7832	0.4768	0.2019	0.6736	0.0017
DE_12_	0.0035	0.3513	0.5723	0.7412	0.4513	0.8923
DE_13_	0.0042	0.2678	0.5898	0.7325	0.4678	0.8725
DE_14_	0.0039	0.3189	0.5943	0.8297	0.4189	0.9736
DE_15_	0.9749	0.7517	0.4124	0.3158	0.4724	0.0073
DE_16_	0.9821	0.7849	0.4239	0.2819	0.4639	0.0081
DE_17_	0.0056	0.3398	0.5523	0.7139	0.4124	0.9651
DE_18_	0.0062	0.2588	0.5845	0.8098	0.4239	0.9723
OE_1_	0.8825	0.7612	0.4287	0.3398	0.6925	0.0056
OE_2_	0.9543	0.1919	0.5183	0.3588	0.6536	0.0062
OE_3_	0.0073	0.3158	0.6135	0.7678	0.4322	0.9279
OE_4_	0.0081	0.2819	0.6243	0.7821	0.4423	0.9153

The government must regularly update its contingency plan for urban infectious disease outbreaks (IC_5_), assess medical resource reserves (IC_4_), and promote public awareness of epidemic resistance. Among the four critical events, IC_4_ significantly decreases casualties (OE_2_). Therefore, it is crucial for priority hospitals and treatment facilities to maintain sufficient medical resource reserves (IC_4_) and enhance government emergency response capacity (IC_6_). A robust government emergency response plan (IC_5_) is pivotal in bolstering public confidence and fostering public trust (OE_3_). Collectively, these four initial conditions can greatly mitigate losses, underscoring the need for adequate emergency preparedness to address the human fatalities and economic ramifications associated with COVID-19.

#### 3.6.2 Analysis of dynamic events

The analysis above indicates that dynamic events related to emergency management include DE_5_, _8_, _11_, _12_, and _14_. The impact of these pivotal events can be similarly assessed, as depicted in Tables 10, 11. The establishment of an emergency command center (DE_8_) significantly reduces economic losses (OE_1_). Implementation of personnel isolation measures (DE_5_) can also mitigate economic loss (OE_2_). While individually, none of these six dynamic events critically affects the four outcome events, their combined effect can be substantial. Therefore, we explored several scenarios (S_6_, _7_, _8_, and _9_).

**Table 10:** Prediction probabilities of the other events in dynamic events analysis.

	S_0_	S_1_	S_2_	S_3_	S_4_	S_5_
DE_1_	0.0017	0.8124	0.6123	0.5823	0.5243	0.5124
DE_2_	0.0019	0.7239	0.6577	0.5977	0.5554	0.5239
DE_3_	0.0027	0.8287	0.6424	0.5824	0.5619	0.5287
DE_4_	0.0030	0.8183	0.6376	0.5676	0.5434	0.5183
DE_5_	0.0000	0.0000	0.0000	1.0000	0.0000	0.0000
DE_6_	0.9883	0.1553	0.2253	0.2587	0.2957	0.3387
DE_7_	0.9875	0.1475	0.2175	0.2683	0.3093	0.3483
DE_8_	0.0000	1.0000	0.0000	0.0000	0.0000	0.0000
DE_9_	0.9923	0.1653	0.2353	0.2787	0.3157	0.3487
DE_10_	0.9725	0.1575	0.2275	0.2583	0.3093	0.3583
DE_11_	0.0000	0.0000	0.0000	0.0000	0.0000	1.0000
DE_12_	0.0000	0.0000	1.0000	0.0000	0.0000	0.0000
DE_13_	0.0044	0.7435	0.6233	0.5733	0.5614	0.5435
DE_14_	0.0000	0.0000	0.0000	0.0000	1.0000	0.0000
DE_15_	0.8825	0.1843	0.2493	0.2686	0.2965	0.3479
DE_16_	0.9543	0.1585	0.2379	0.2673	0.3001	0.3215
DE_17_	0.9736	0.1375	0.2395	0.2780	0.3126	0.3562
DE_18_	0.0000	0.8087	0.6224	0.5624	0.5419	0.5087
OE_1_	0.9749	0.1234	0.2179	0.2987	0.3056	0.3341
OE_2_	0.9821	0.1219	0.2469	0.2587	0.2934	0.3490
OE_3_	0.0012	0.8383	0.6576	0.5876	0.5634	0.5383
OE_4_	0.0015	0.7439	0.6777	0.6177	0.5754	0.5439

**Table 11:** Prediction probabilities of the other events in dynamic events analysis.

	S_6_	S_7_	S_8_	S_9_	S_10_
DE_1_	0.8823	0.7824	0.9543	0.9823	0.8249
DE_2_	0.8577	0.7639	0.9554	0.9977	0.8039
DE_3_	0.8424	0.7787	0.9419	0.9984	0.8187
DE_4_	0.8676	0.7883	0.9434	0.9776	0.8383
DE_5_	0.0000	0.0000	1.0000	1.0000	1.0000
DE_6_	0.0187	0.0953	0.0087	0.0017	0.0553
DE_7_	0.0283	0.0975	0.0083	0.0013	0.0575
DE_8_	1.0000	0.0000	0.0000	1.0000	0.0000
DE_9_	0.0387	0.1053	0.0092	0.0016	0.0753
DE_10_	0.0249	0.1089	0.0073	0.0011	0.0675
DE_11_	0.0000	1.0000	1.0000	1.0000	1.0000
DE_12_	1.0000	0.0000	0.0000	1.0000	0.0000
DE_13_	0.8733	0.7635	0.9314	0.9733	0.8235
DE_14_	0.0000	1.0000	1.0000	1.0000	0.0000
DE_15_	0.0190	0.0903	0.0086	0.0019	0.0513
DE_16_	0.0173	0.0915	0.0081	0.0011	0.0589
DE_17_	0.0274	0.1134	0.0079	0.0021	0.0502
DE_18_	0.8224	0.7587	0.9219	0.9784	0.7987
OE_1_	0.0356	0.1289	0.0072	0.0046	0.0563
OE_2_	0.0279	0.1178	0.0081	0.0058	0.0597
OE_3_	0.8868	0.8045	0.9475	0.9712	0.8541
OE_4_	0.8723	0.7859	0.9598	0.9942	0.8241

In S_6_, where DE_12_ = DE_8_ = 1, DE_5_ = DE_14_ = DE_11_ = 0, personnel movement is restricted, and an emergency command center is established. This configuration significantly reduces casualties (OE_2_) and somewhat mitigates economic losses (OE_1_). Effective rescue efforts and movement restrictions in the COVID-19-affected area of Wuhan are crucial for minimizing casualties, economic losses, and fostering public trust (OE_4_). Government initiatives to guide public opinion, disseminate information about infectious diseases through media, and promote awareness about hazards and preventive measures play a crucial role in alleviating social panic (OE_4_). In scenario 7 (S_7_), economic losses (OE_1_) are notably reduced, but the decrease in casualties (OE_1_) is insignificant and has minimal impact on enhancing public trust (OE_3_). In scenario 8 (S_8_), where DE_12_ = DE_8_ = 0 and DE_5_ = DE_11_ = DE_14_ = 1, there is a significant decrease in economic losses (OE_1_), a notable increase in public trust (OE_4_), and a considerable decrease in casualties (OE_2_).

## 4 Applications

### 4.1 Scenario reasoning for emergency management of COVID-19 in Wuhan, China

Analyzing the epidemic’s progression reveals the critical timeline of anti-epidemic events subsequent to the COVID-19 outbreak in Wuhan, detailed in Table 13. Initially, Wuhan Jinyintan Hospital faced shortages in essential resources for infectious disease control, including protective equipment such as medical gowns, masks, and goggles (39). In December 2019, Wuhan reported pneumonia cases attributed to a novel coronavirus strain, distinct from the 2003 SARS outbreak, prompting speculation about a new coronavirus variant (40). Consequently, IC_1_ = IC_2_ = IC_3_ = 1, and IC_4_ = 0. Despite advancements in infectious disease management, Wuhan hospitals’ routine infection prevention measures proved insufficient for novel viruses (41). While Wuhan regularly conducted outbreak drills for common infectious diseases, these preparations lacked adequacy for highly transmissible viruses, undermining their response capabilities. Public self-protection measures were initially deficient, with limited access to clear guidance exacerbating the situation (42, 43). Local governments struggled with public communication, lacking comprehensive plans for engagement, although public trust in government instructions remained intact. Given the novelty of the coronavirus, its initial emergence saw no available vaccine (44). To summarize, the initial event probabilities are IC_5_ = IC_6_ = IC_7_ = IC_8_ = IC_10_ = 0 and IC_9_ = 1.

Once dynamic events occur, they have a probability of 1. At the onset of the COVID-19 outbreak, numerous cases emerged both within and outside Hubei Province, with infections also reported in other countries. This situation led to varying degrees of panic among the population (45). Concurrently, a plethora of rumors circulated on the internet and social media platforms, disseminating information about infectious diseases, primarily on platforms like WeChat and Weibo (46). Although routine emergency medical supplies were swiftly distributed to organizations and individuals in need, shortages quickly became apparent (47). Meanwhile, the outbreak rapidly proliferated, spreading to other provinces and cities across China (48). To curb the spread of COVID-19, China enforced traffic control measures in Wuhan city on January 23, 2020, resulting in DE_7_ = DE_9_ = DE_10_ = DE_17_ = DE_6_ = 1, DE_18_ = 0. However, mass production of the vaccine remains unfeasible in the short term (49), and sporadic instances occur where local authorities withhold information about the epidemic’s status from the public (50).

### 4.2 Results of cross-impact analysis

The probabilities of events in the six scenarios are presented in Table 12, derived from the preceding formula. In accordance with the temporal order of key events, DE_8_ = DE_12_ = DE_5_ = DE_14_ = DE_11_ = 1 was established, as illustrated in Table 14. Additionally, leveraging the timeline provided in Table 13, we inferred the epidemic spread scenario post-COVID-19 outbreak in Wuhan using seven steps (51), as depicted in Table 14.

**Table 12:** Prediction probabilities of the other events under each scenario.

	S_0_	S_1_	S_2_	S_3_	S_4_	S_5_
IC_1_	1	1	1	1	1	1
IC_2_	1	1	1	1	1	1
IC_3_	1	1	1	1	1	1
IC_4_	0	0	0	0	0	0
IC_5_	0	0	0	0	0	0
IC_6_	0	0	0	0	0	0
IC_7_	0	0	0	0	0	0
IC_8_	0	0	0	0	0	0
IC_9_	1	1	1	1	1	1
IC_10_	0	0	0	0	0	0
DE_1_	0.788	0.7655	0.8212	0.8542	0.8679	0.9013
DE_2_	0.878	0.8524	0.8449	0.8721	0.9266	0.9657
DE_3_	0.614	0.5679	0.6241	0.6412	0.7987	0.8977
DE_4_	0.624	0.5972	0.6897	0.7825	0.8554	0.9019
DE_5_	0.554	0.5143	0.6045	0.8522	0.8769	0.9212
DE_6_	0.998	1	1	1	1	1
DE_7_	0.969	1	1	1	1	1
DE_8_	0.875	0.8539	1	1	1	1
DE_9_	0.97	1	1	1	1	1
DE_10_	0.991	1	1	1	1	1
DE_11_	0.977	1	1	1	1	1
DE_12_	0.771	0.7312	0.7613	1	1	1
DE_13_	0.582	0.5598	0.6712	0.7012	1	1
DE_14_	0.739	0.7111	0.7688	0.8099	1	1
DE_15_	0.767	0.7933	0.6012	0.5691	0.4115	0.3011
DE_16_	0.869	0.8721	0.6012	0.5576	0.3862	0.2512
DE_17_	0.713	0.6982	0.8211	0.8622	0.8723	1
DE_18_	0.892	0	0	0	0	0
OE_1_	0.987	0.9921	0.9666	0.9489	0.9415	0.9233
OE_2_	0.979	0.9867	0.8791	0.8344	0.7562	0.6725
OE_3_	0.841	0.8213	0.8612	0.8879	0.9012	0.9588
OE_4_	0.152	0.1091	0.7729	0.7811	0.8466	0.9065

**Table 13:** Timeline of major rescue events after COVID-19 in Wuhan.

Time	Events
Dec 27, 2019	The hospital reported a case of unexplained pneumonia to the Jianghan District CDC.
Dec 30	The National Health and Wellness Commission was informed of this and immediately organized research and prompt action.
Dec 31	27 cases were found, prompting the public to avoid public places and crowded places, and to wear masks when going out.
Jan 1, 2020	An outbreak response leadership team is established. CDC and Chinese Academy of Medical Sciences receive cases and immediately carry out pathogen identification.
Jan 3	Further pathogen identification China regularly and proactively informs the World Health Organization of outbreak information.
Jan 4	Develop a workbook for medical treatment of viral pneumonia of known cause and reach a consensus with the CDC for close liaison.
Jan 5	The World Health Organization informs about the cases of unexplained pneumonia in Wuhan.
Jan 7	Successful isolation of a novel coronavirus strain by the Chinese CDC.
Jan 9	The pathogen was initially determined to be a novel coronavirus.
Jan 10	The National Health and Wellness Commission shares information on the genome sequence of the new coronavirus with the World Health Organization.
Jan 15	Released the first version of the treatment, prevention and control protocol for pneumonia with novel coronavirus infection.
Jan 17	The National Health and Wellness Commission sent seven supervisory teams to localities to guide the prevention and control of the epidemic.
Jan 18	Release the second version of the treatment protocol for pneumonia with novel coronavirus infection.
Jan 19	Organized a high-level expert group on prevention and control to rush to Wuhan City for a field study on the prevention and control of the outbreak. Clarify that human-to-human transmission of the new coronavirus is occurring.
Jan 23	Airports and train stations are temporarily closed for departures from Wuhan. Provinces across the country activate provincial-level emergency response for major public health emergencies one after another.
Jan 24	National medical teams and public health personnel are mobilized from various regions and the military to assist Hubei Province and Wuhan City.
Jan 25	Sent steering teams to Wuhan and other areas with serious outbreaks to promote strengthening of front-line prevention and control efforts.
Jan 26	Extend the 2020 Spring Festival holiday and postpone the opening of colleges, universities, primary and secondary schools, and kindergartens around the country.
Jan 27	The central steering team is stationed in Wuhan to comprehensively strengthen guidance and supervision of the frontline prevention and control of the epidemic.

**Table 14:** Wuhan COVID-19 situational rehearsal setup.

Step	Scenario
Step_0_	IC_1_ = IC_2_ = IC_3_ = IC_9_ = 1 IC_4_ = IC_5_ = IC_6_ = IC_7_ = IC_8_ = IC_10_ = 0,
Step_1_	DE_7_ = DE_9_ = DE_10_ = DE_11_ = DE_6_ = 1, DE_18_ = 0
Step_2_	DE_13_ = 1
Step_3_	DE_8_ = DE_14_ = 1
Step_4_	DE_12_ = 1
Step_5_	DE_17_ = 1

**Figure 6:**
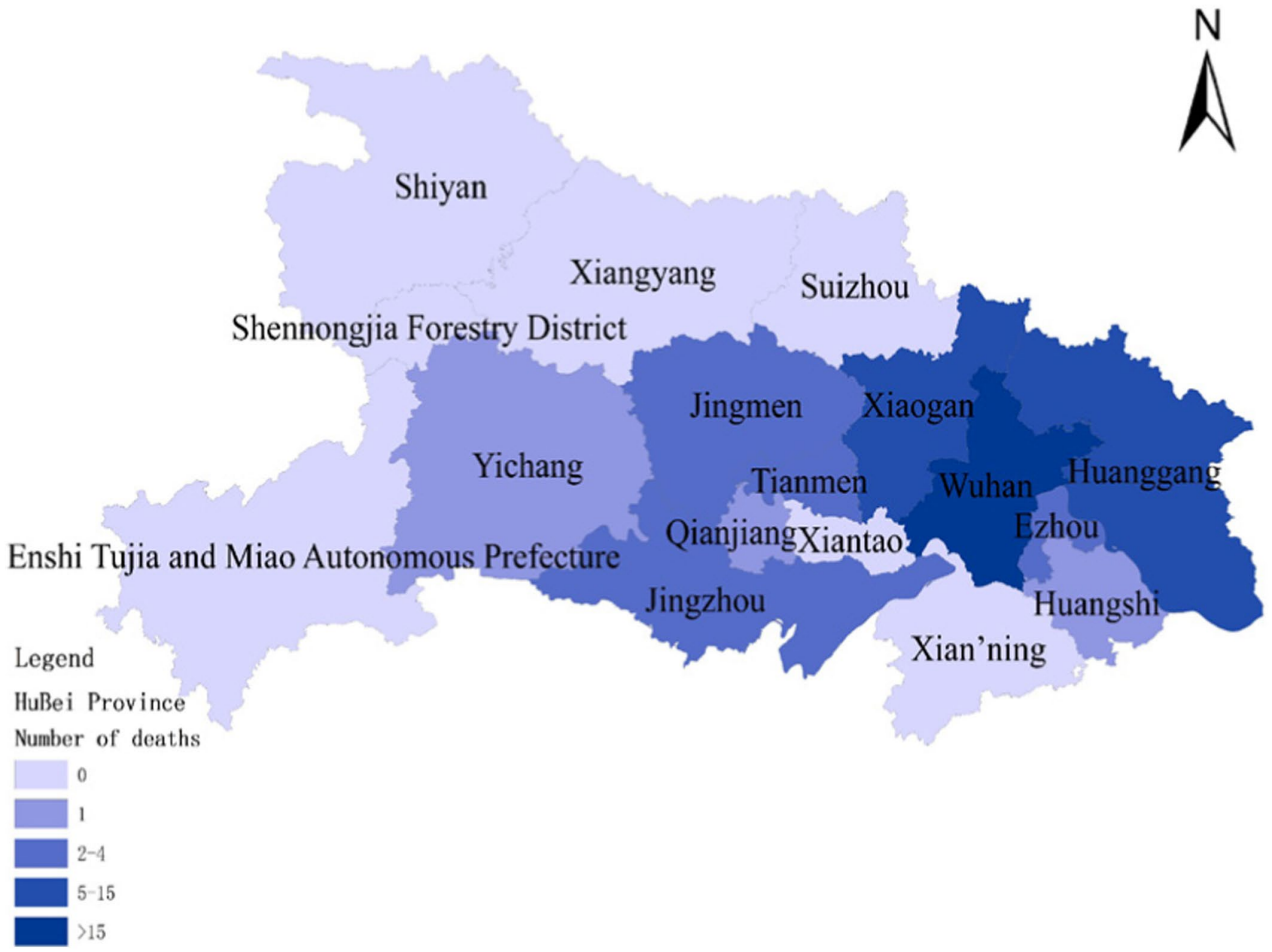
Cumulative deaths due to COVID-19 on January 30.

**Figure 7:**
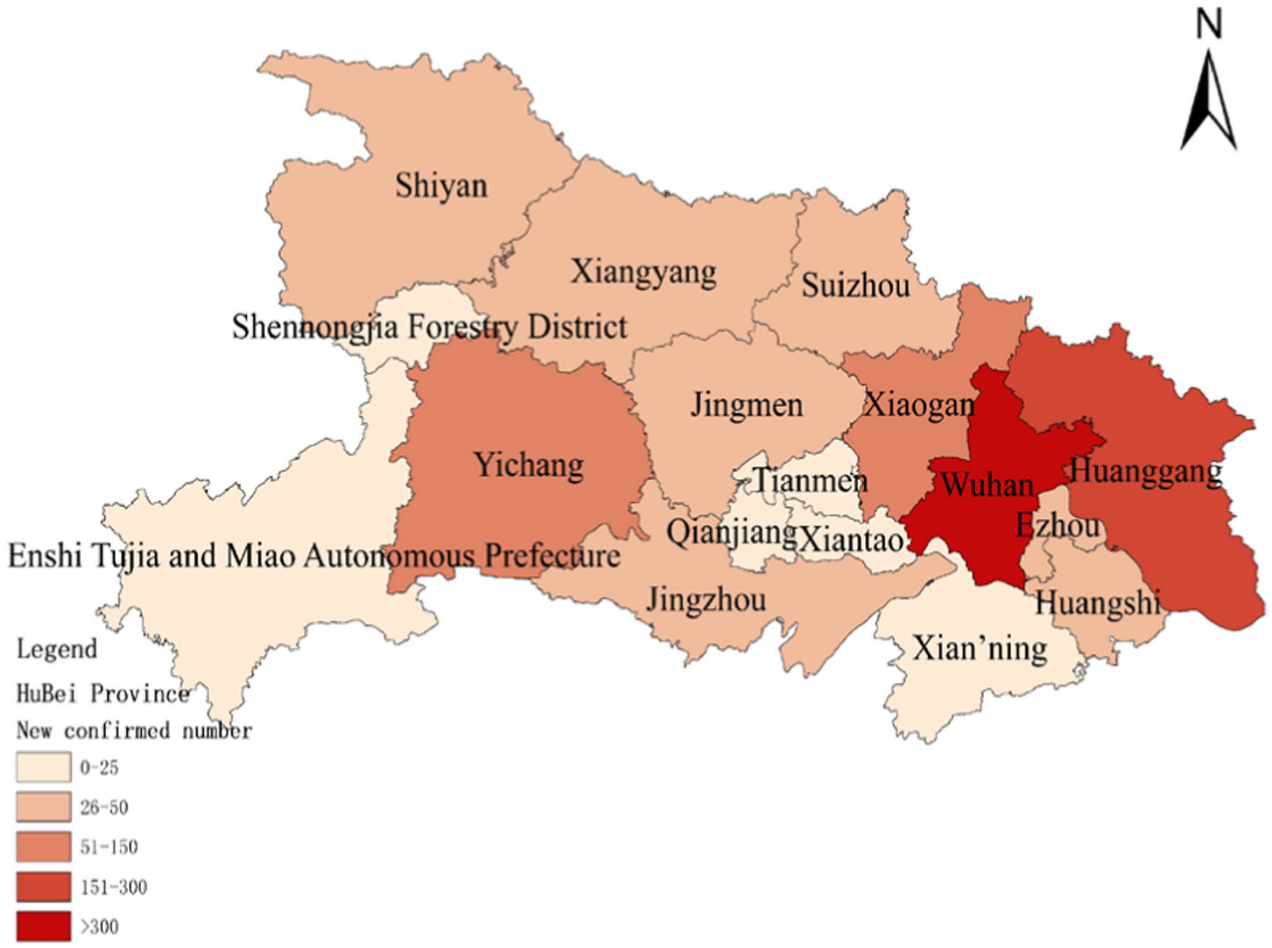
Number of new confirmed COVID-19 diagnoses on January 30.

**Figure 8:**
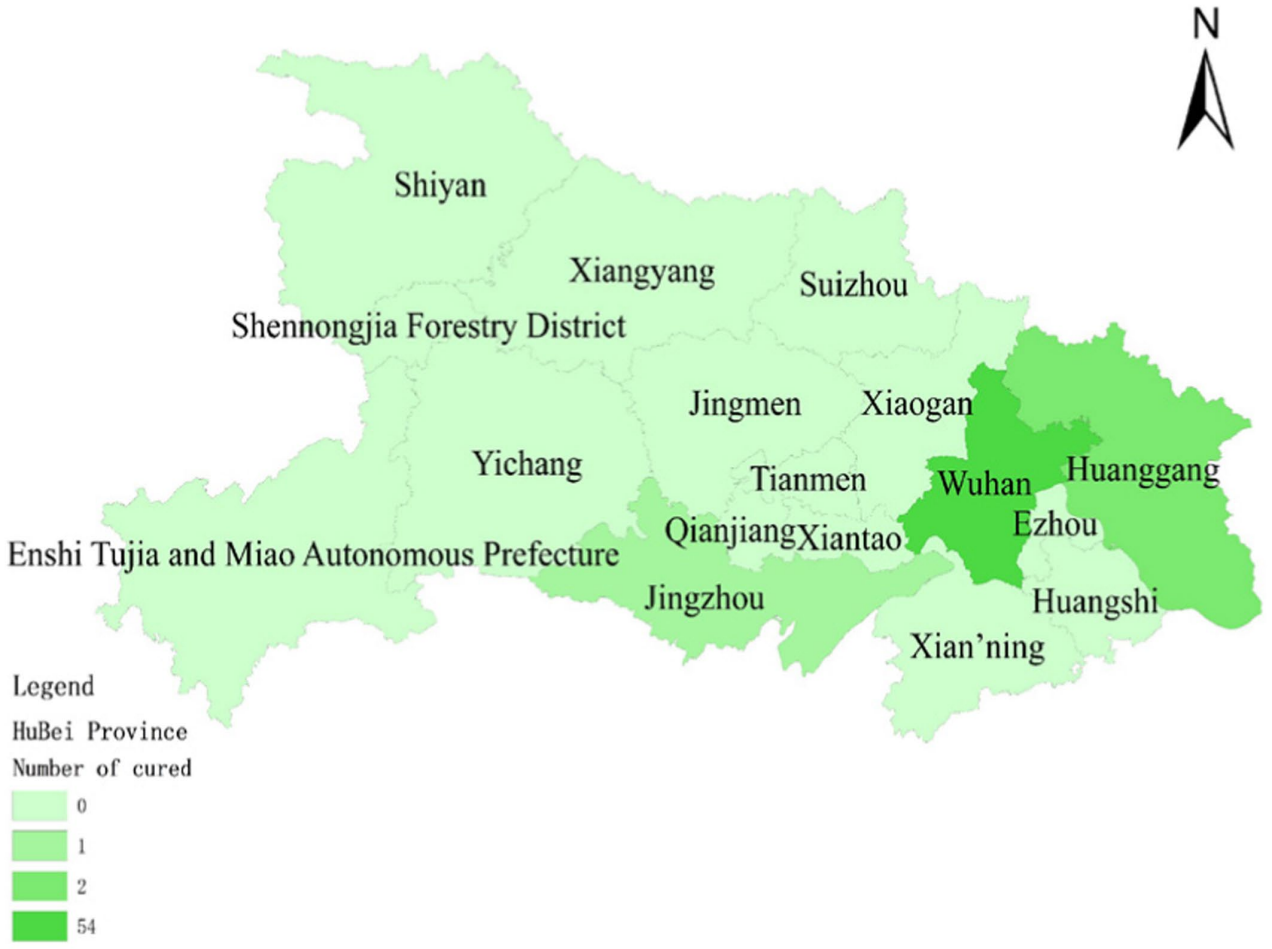
Number of people cured after having COVID-19 on January 30.

To assess the model’s accuracy, the predicted outcomes were juxtaposed with the actual COVID-19 situation in Wuhan. As of March 10, 2020, at 24:00, Hubei Province had reported a cumulative total of 67,773 confirmed cases, 49,056 recoveries, and 3,046 deaths. Following the initial onset of COVID-19, there was a notable surge in mortality and daily new cases, accompanied by a poor recovery rate, as depicted in Figures 6–8.

**Figure 9:**
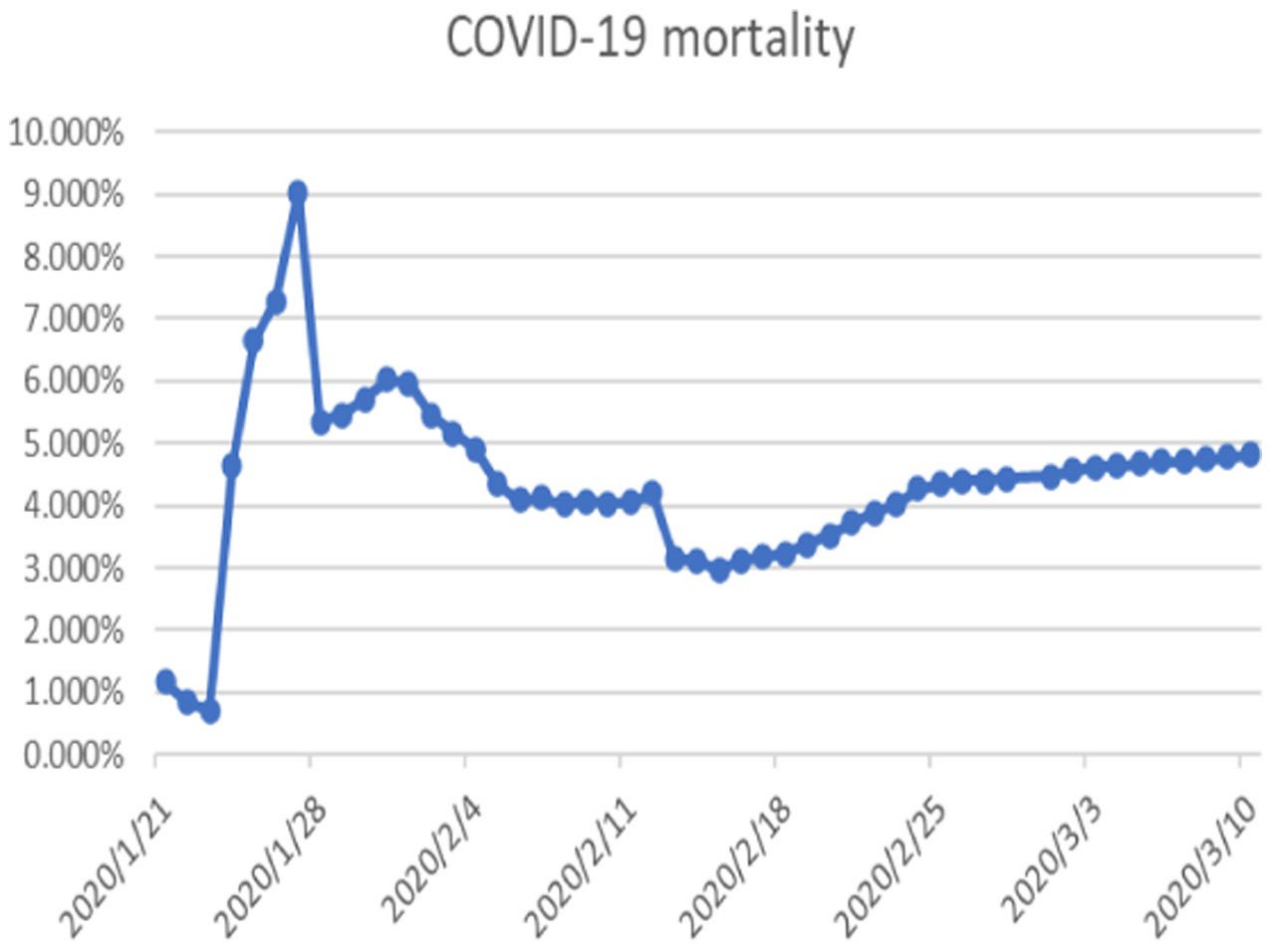
COVID-19 mortality rate.

**Figure 10:**
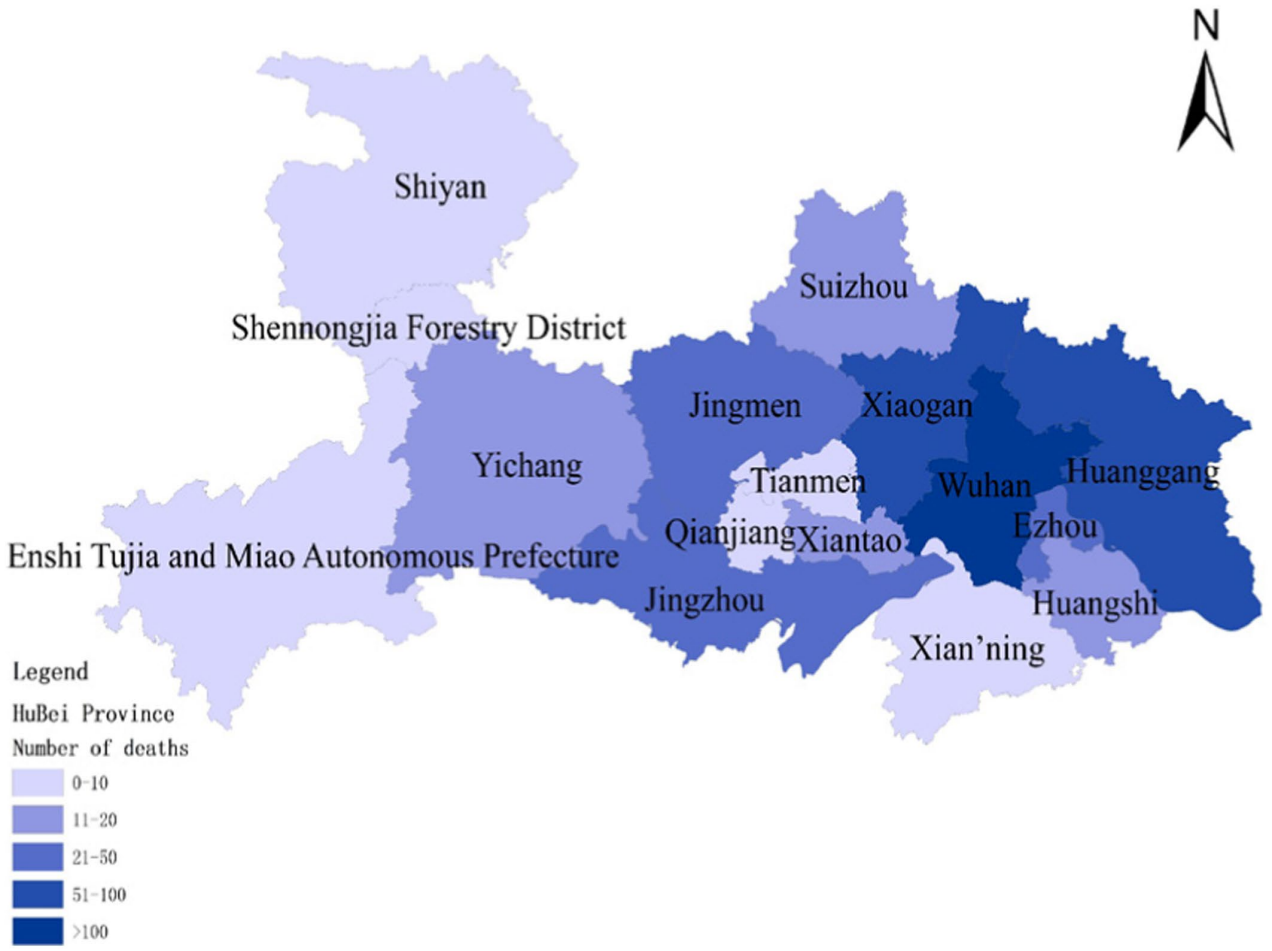
Cumulative deaths due to COVID-19 on February 14.

**Figure 11:**
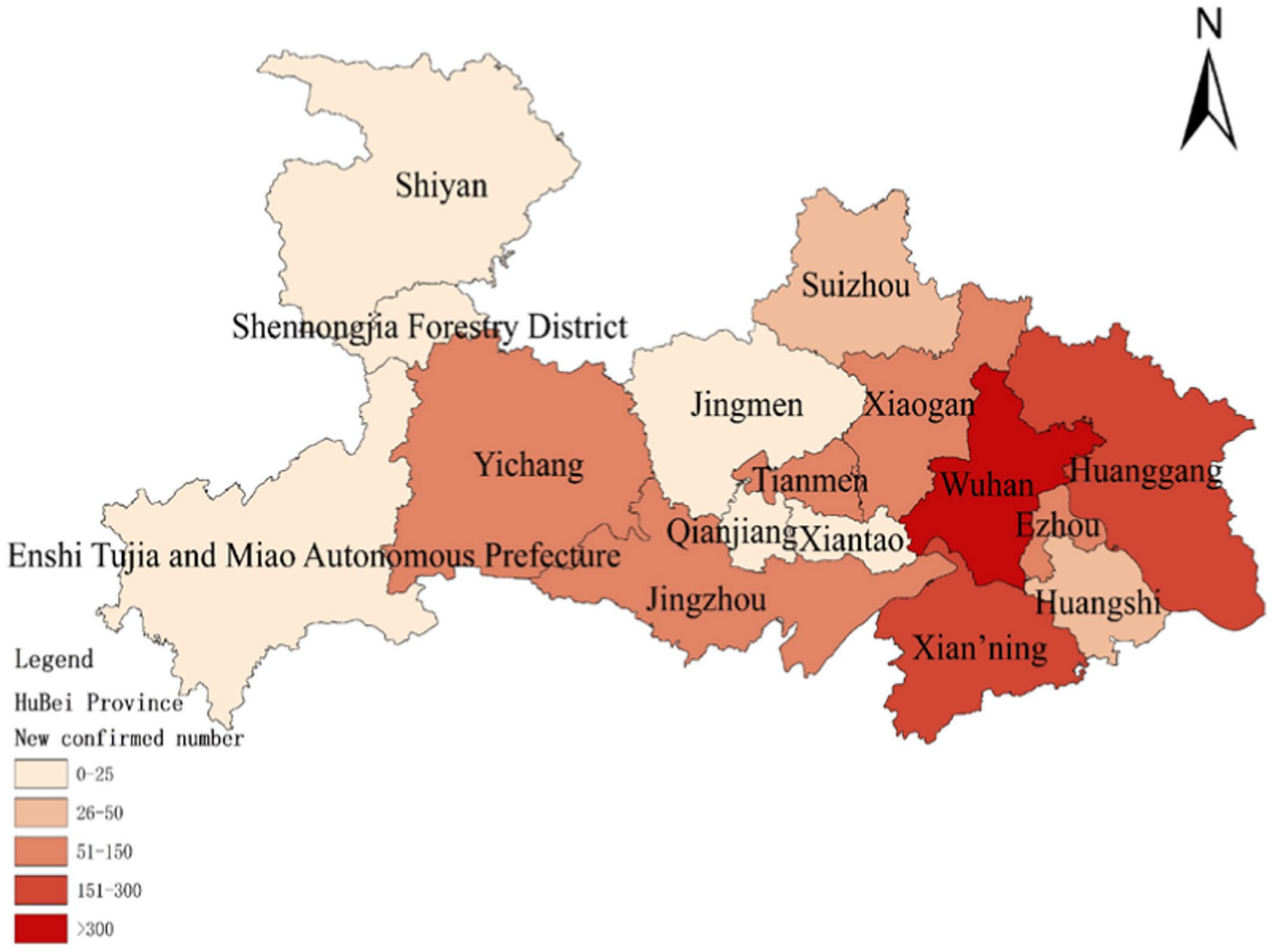
Number of new confirmed COVID-19 diagnoses on February 14.

**Figure 12:**
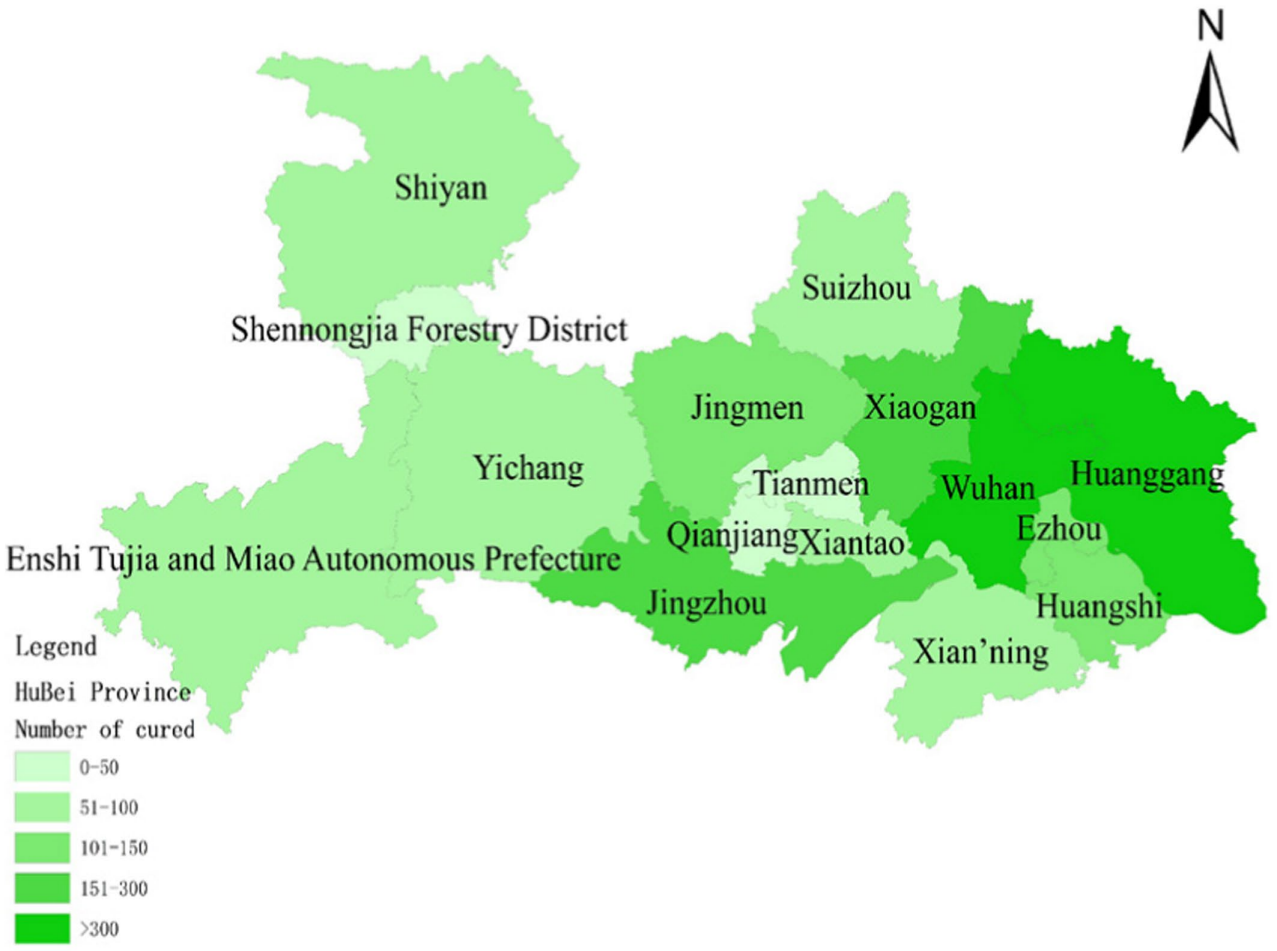
Number of people cured after having COVID-19 on February 14.

**Figure 13:**
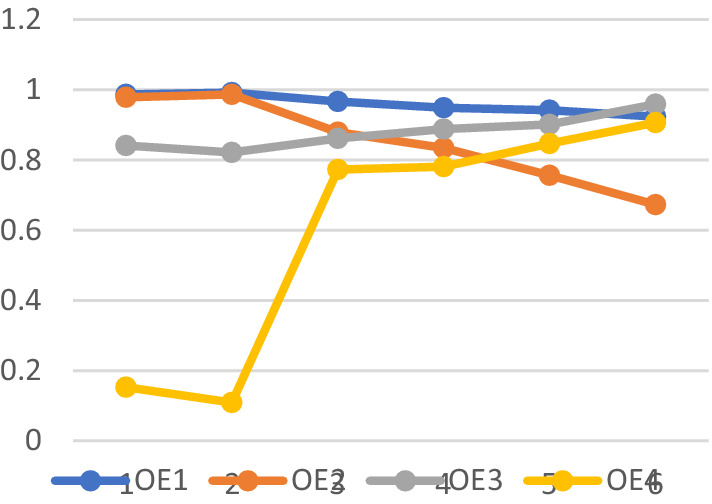
The trend of the prediction probabilities of four outcome events.

The probability of human casualties (OE_2_) surged from 1.163% to nearly 9%, as illustrated in Figure 9. As Chikyu Wuhan intensified its anti-epidemic efforts, the COVID-19 mortality rate swiftly dropped from 9.026% on January 27 to 5.346% on January 28. In Wuhan, the number of new cases exhibited the initial signs of decline by 2.14, marking the beginning of a downturn in cumulative deaths, while the tally of recoveries continued to rise, as depicted in Figures 10–13.

Subsequent mortality rates continued to decrease and stabilized below 5%, indicating that the epidemic was largely under control by early March, as evidenced in the attached video. The projected trend aligns with the actual situation outlined in the statistical report.

COVID-19 resulted in damages exceeding $1.1 trillion. Despite substantial donations and supplies raised by the government, they had minimal impact on offsetting the substantial losses. The probability of economic loss (OE_1_) consistently hovered near 100% with minimal fluctuations, mirroring the actual scenario. Emergency response measures following the COVID-19 outbreak had limited success in mitigating economic losses, emphasizing the need for stronger focus on emergency preparedness. The Chinese government is lauded for its swift emergency response to the COVID-19 outbreak. The central government promptly dispatched medical personnel to affected regions within 4 days of the outbreak. Various departments actively gathered and validated valuable data, while the National Health Commission promptly disseminated the latest updates to the public. The prompt dissemination of authoritative information alleviated public panic and anxiety to some extent, garnering praise domestically and internationally. Following COVID-19, the probability of public trust (OE_4_) decreased to nearly 10%, reflecting the actual circumstances. Public trust notably surged after the government’s swift relief measures. The government efficiently shaped public opinion and promptly disclosed outbreak information, markedly reducing social panic and enhancing public trust. The probability of public trust was highest upon the arrival of rescue teams and supplies in Wuhan, coupled with the prompt distribution of emergency medical supplies to those in need. The findings suggest that the government actively quelled public panic and fostered public trust, with the establishment of the emergency command center being pivotal. Nonetheless, in the initial scenario (step_0_), the probabilities of both outcome events reached very high values, underscoring the substantial impact of the lack of emergency preparedness on outcomes resulting in severe losses. The simulation results better align with the actual scenario, as depicted in Figure 12.

The emergency response to the COVID-19 outbreak in Wuhan was swift and effective. However, weak emergency preparedness could still lead to significant casualties and substantial economic losses. Our simulation aims to create dynamic scenarios for potential urban infectious disease outbreaks. Developing scenario-based contingency plans for such outbreaks can assist decision-makers in analyzing potential scenarios during the COVID-19 pandemic and predicting the impact and consequences of various actions they may take. The simulation includes four scenarios: IC_4_ = 1, IC_5_ = IC_7_ = 0; IC_5_ = 1, IC_4_ = IC_7_ = 0; IC_7_ = 1, IC_4_ = IC_5_ = 0; IC_4_ = IC_7_ = IC_5_ = 1. The remaining parameters mirror those of the Wuhan COVID-19 outbreak.

**Figure 14:**
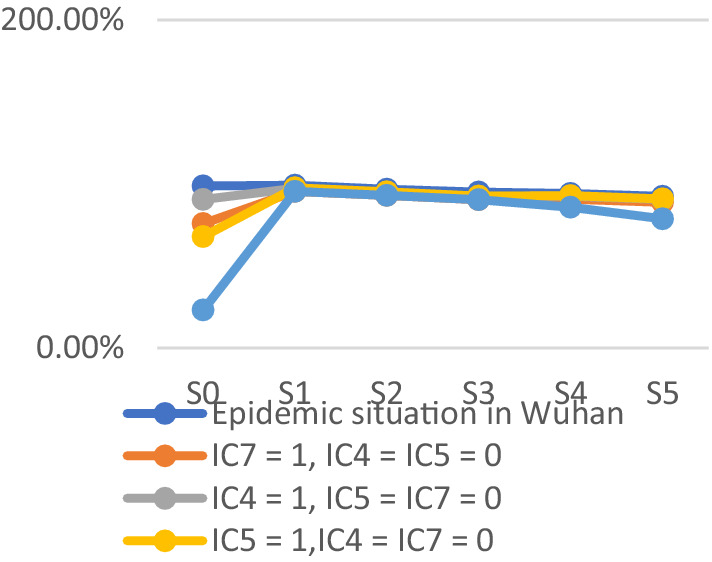
Economic loss.

**Figure 15:**
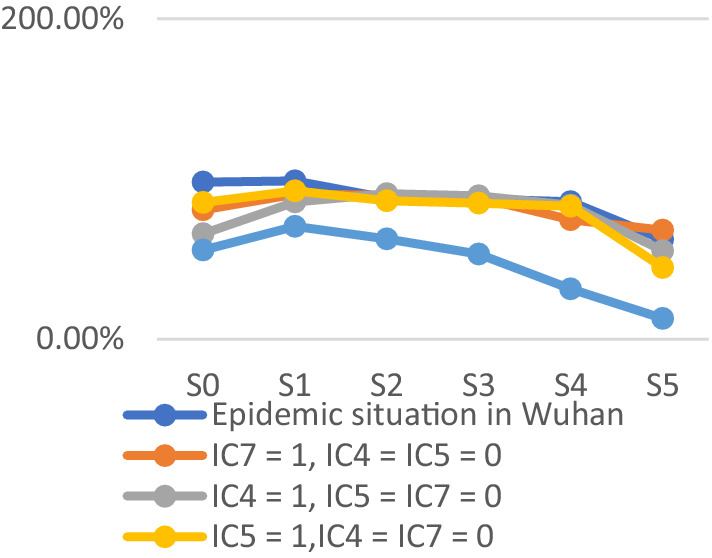
Personnel death.

**Figure 16:**
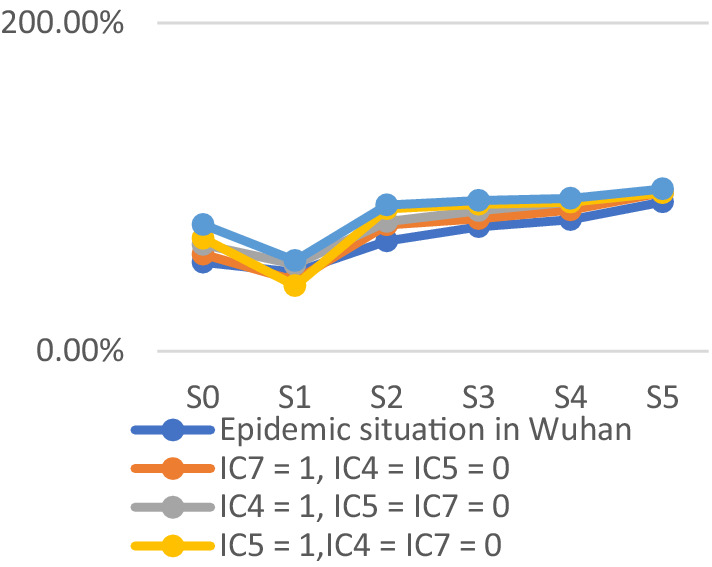
Infectious diseases are under control.

**Figure 17:**
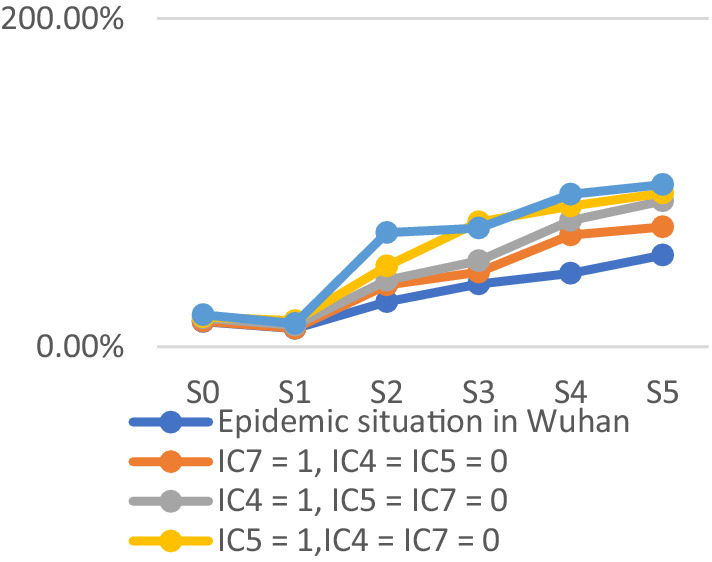
Public trust.

The probabilities of four outcome events - human casualties, economic losses, disease control, and public trust under different scenarios - are illustrated in Figures 14–17, respectively. Figure 15 demonstrates that government communication capacity (IC_8_) positively impacts reducing economic losses. However, recovering from significant economic losses caused by COVID-19 proves challenging. Figure 15 also highlights the critical roles of medical resource reserves (IC_4_) and public self-protection capabilities (IC_7_) in mitigating human casualties, alongside the positive effect of government contingency plans (IC_5_). Factors such as public panic (DE_9_), rumor spreading (DE_10_), disease transmission (DE_11_), leadership discord in releasing information (DE_6_), and information leakage (DE_7_) significantly increase the likelihood of human casualties (Step_2_). Conversely, Figure 16 indicates that sufficient government contingency plans (IC_5_) and medical resource reserves (IC_4_) can expedite disease control, albeit with the risk of heightened social panic (Step_2_) due to disease spread. Nonetheless, effective emergency preparedness and robust government contingency plans swiftly mitigate public panic and foster public trust, as depicted in Figure 17. Therefore, several recommendations emerge, emphasizing the pivotal role of emergency preparedness in guiding positive responses from both government and the public to COVID-19. Simultaneously, efforts should focus on minimizing associated risks and losses. Easy access to transportation facilities may exacerbate disease spread, necessitating contact isolation measures for transit travelers in managing urban disease outbreak emergencies. Moreover, addressing rumor spreading and information leakage warrants increased governmental attention, along with the development of comprehensive emergency response measures and extensive public education. Frontline medical personnel’s rescue efforts and adequate medical resources are crucial for saving lives, facilitating easier epidemic control and ensuring prompt deployment of medical resources. Given the economic challenge posed by urban disease outbreaks, hospitals’ medical facility performance and local hospitals’ rescue efforts are particularly crucial. Finally, active public opinion guidance and timely, effective dissemination of epidemic-related information are indispensable for reducing social panic and enhancing public trust.

## 5 Discussion and conclusion

### 5.1 Discussion

Emergency medical supplies directly impact casualties by being delivered to organizations and people in need, aligning with Tokalić and Viđak’s study (12). Stockpiling medical resources and establishing an emergency command center are crucial priorities in emergency preparedness to prevent mass casualties. Several studies have explored the interplay between infectious diseases and other social crises or urban infrastructure (52, 53). For instance, Jakariya et al. developed an integrated wastewater-based epidemiological system to detect COVID-19 prevalence (54), while Islam et al. emphasized the importance of COVID-19 antibody studies (55). However, this study focuses on the early stages of infectious disease outbreaks and does not include antibodies as a key factor due to their development time (56). Vulnerability to social and political factors may undermine COVID-19 emergency management efforts, consistent with previous findings (57). Global mortality trends of COVID-19 reflect inadequate preparedness worldwide (58). Rahman et al.’s systematic evaluation approach offers valuable insights for this study, particularly in response measures for Southeast and South Asia (59). However, most studies only address single points in emergency management, such as evacuation or healthcare responses (60, 61). Unlike Brooks et al.’s study, this paper examines the dynamic interaction between event sets and constructs a scenario model for analyzing COVID-19 emergency management in Wuhan (7). It presents a hypothetical urban outbreak response scenario based on COVID-19 occurrences, highlighting the impact of critical events on outcomes and using directed graphs to illustrate event relationships. The leaders’ reluctance to share information and healthcare workers’ non-cooperation trigger other dynamic events, emphasizing the importance of emergency preparedness in epidemic response. The model proposed in this research enriches the future pandemic response framework proposed by Amir Khorram-Manesh et al. (62).

### 5.2 Conclusion

This paper convenes an expert panel of emergency management specialists and Wuhan COVID-19 responders, leveraging insights from prior infectious disease outbreaks like H_1_N_1_ and SARS (5). The panel compiles a comprehensive array of critical events pertaining to emergency management decisions. Utilizing the Delphi method, a consistent causality estimation matrix is established among these events. Following the creation of a cross-impact matrix, the most impactful events, representing the top 5 and 15%, are visually depicted. Initially, the model facilitates multiple scenario analyses to identify crucial contingencies. Subsequently, it discerns correlations between actual events to delineate outcome trends stemming from varied courses of action. Emergency management initiatives that synergistically influence one another form micro-sets, enabling the dissection of their impact on human casualties and economic losses. Scenario simulations derived from diverse emergency management strategies prove more scientifically robust than single-factor approaches (19). Concurrently, this study corroborates the adverse effects of rumors and information leakage on infectious disease containment (34). Restricting people’s movement significantly curtails casualties, while the swift depletion of medical supplies and nationwide economic shutdowns exacerbate economic losses. Although rapid economic recovery from infectious disease-induced losses remains challenging, a well-crafted emergency plan serves as a cornerstone for restoring public confidence and bolstering trust. Enhanced public awareness of effective epidemic control measures and self-protection strategies expedites disease coping mechanisms. However, casualties, information leaks, and rumors can escalate public panic, underscoring the need for proactive guidance through essential medical interventions and authoritative dissemination of disease-related information by governmental bodies. The findings underscore the imperative of emergency preparedness in mitigating severe economic losses, casualties, and societal ramifications precipitated by infectious diseases. During the primary stage of emergency response, concerted rescue efforts must collaborate to achieve optimal outcomes. Simulation outcomes demonstrate that the effective implementation of multiple emergency management measures significantly diminishes the probability of infectious disease-related damage. The analysis underscores the pivotal role of establishing an emergency command center and the proactive governmental involvement in assuaging public apprehension and fostering trust. Prioritizing the isolation and monitoring of individuals exposed to infectious diseases emerges as a paramount objective in emergency management, effectively curbing disease transmission and mitigating casualties and economic losses. Drawing from real-world epidemic occurrences and scenario simulations, this paper enables the assessment of the efficacy of critical emergency management measures, the identification of pivotal events for future epidemic combat, and the formulation of targeted recommendations to ameliorate epidemic-induced losses and refine future emergency management protocols.

## Data availability statement

The original contributions presented in the study are included in the article/supplementary material, further inquiries can be directed to the corresponding authors.

## Author contributions

PY: Writing – original draft, Writing – review & editing. HL: Writing – original draft. XD: Writing – review & editing.
